# Simultaneous Analysis of Secondary Structure and Light Scattering from Circular Dichroism Titrations: Application to Vectofusin-1

**DOI:** 10.1038/srep39450

**Published:** 2016-12-22

**Authors:** Louic S. Vermeer, Arnaud Marquette, Michel Schoup, David Fenard, Anne Galy, Burkhard Bechinger

**Affiliations:** 1 University of Strasbourg, Institute of Chemistry, CNRS, UMR 7177, Membrane Biophysics and NMR group, 1 rue Blaise Pascal, 67000 Strasbourg, France; 2 Généthon, INSERM UMR S951, 1 rue de l’Internationale, F-91002 Evry, France

## Abstract

Circular Dichroism data are often decomposed into their constituent spectra to quantify the secondary structure of peptides or proteins but the estimation of the secondary structure content fails when light scattering leads to spectral distortion. If peptide-induced liposome self-association occurs, subtracting control curves cannot correct for this. We show that if the cause of the light scattering is independent from the peptide structural changes, the CD spectra can be corrected using principal component analysis (PCA). The light scattering itself is analysed and found to be in good agreement with backscattering experiments. This method therefore allows to simultaneously follow structural changes related to peptide-liposome binding as well as peptide induced liposome self-association. We apply this method to study the structural changes and liposome binding of vectofusin-1, a transduction enhancing peptide used in lentivirus based gene therapy. Vectofusin-1 binds to POPC/POPS liposomes, causing a reversal of the negative liposome charge at high peptide concentrations. When the peptide charges exactly neutralise the lipid charges on both leaflets reversible liposome self-association occurs. These results are in good agreement with biological observations and provide further insight into the conditions required for efficent transduction enhancement.

Circular dichroism (CD) spectroscopy is a widely used method to study protein structure^1^,^2^,^3^. If good quality spectra are obtained, they can be decomposed into their constituent spectra and the protein secondary structure quantified^4^,^5^,^6^,^7^. When the structural changes are related to membrane binding, an association isotherm can be determined from a titration experiment with liposomes^8^,^9^,^10^,^11^. Light scattering caused by protein or liposome self-association can severely distort the spectra and prevent a quantitative analysis of the secondary structure, usually leading to the underestimation of the *α*-helical content. If light scattering is only caused by the presence of liposomes the subtraction of control curves is sufficient^8^, but if peptide-induced liposome self-association occurs, control curves of a titration without peptide cannot be used for such a correction.

In this paper we show that if the cause of the light scattering is independent from the peptide structural changes, the CD spectra can be corrected for said scattering using principal component analysis (PCA)^12^. Although PCA decomposition itself and its use to analyse titration data are well established^13^,^14^, the application presented here to correct and analyse light scattering in CD spectroscopy is useful to extract the maximum amount of information from such an experiment, particularly in the context of automated screening approaches. We demonstrate that this correction leads to a better estimate of the secondary structure content. In addition, we analyse the light scattering itself and show that it is in good agreement with static and dynamic light scattering data measured independently using backscatter. Finally, we apply this method to study the structural changes of the vectofusin-1 peptide when it binds to liposomes and give a detailed analysis of the molecular basis for the peptide-induced liposome self-association which is in good agreement with biological observations on this transduction enhancing peptide used in gene therapy^15^,^16^.

Besides the case demonstrated here we have observed distortions in CD spectra due to light scattering in a multitude of studies and expect spectral decomposition for analysis and removal of light scattering to be widely applicable, for example in studies of antimicrobial peptides interacting with liposomes^17^ or entire bacteria^18^, studies of peptide self-association in the presence of phosphate or other divalent ions^19^, investigations of nucleic-acid delivery peptides that cause DNA condensation^20^, the interaction of transduction enhancing peptides with liposomes or intact viruses, the characterisation and screening of peptide and protein samples for x-ray crystallography, and the optimisation of bicelle and nanodisc samples for NMR spectroscopy. Studies of liposome-induced fibril formation by amyloid and amyloid-like proteins and peptides may also benefit, as long as the light scattering is not correlated with the structural changes.

## Results

### Circular Dichroism

**Figure 1:**
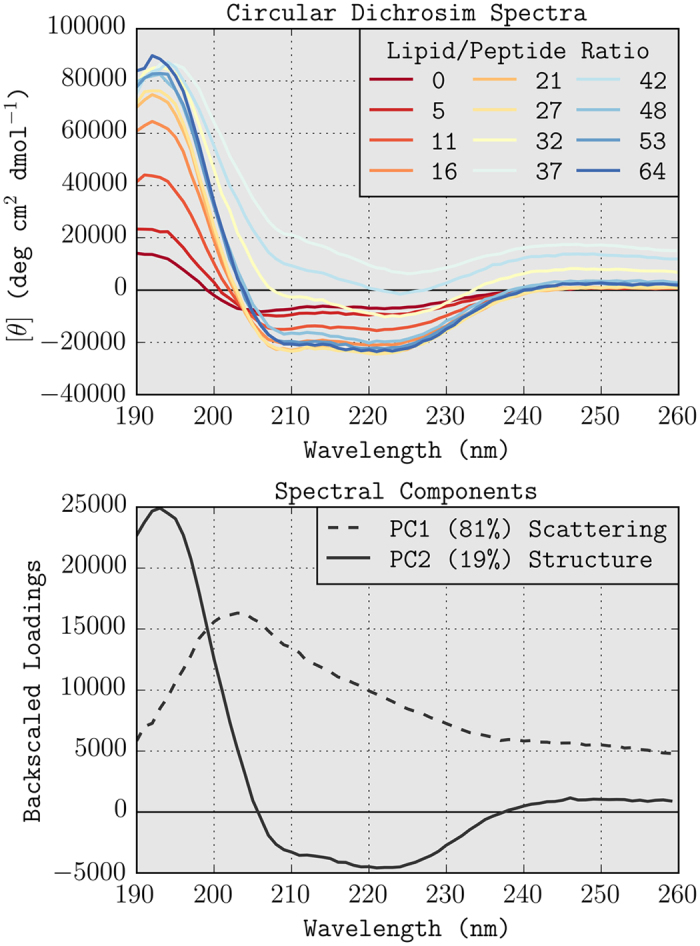
(**A**) Titration of vectofusin-1 with POPC/POPS liposomes. Spectra are corrected with control curves of liposomes only but peptide-induced liposome aggregation causes some of the spectra to be severly affected by light scattering. (**B**) Spectral components as determined by principal component analysis. The CD spectra are fully described by two components that we interpret as light scattering (PC1) and peptide structural changes (PC2).

**Figure 2:**
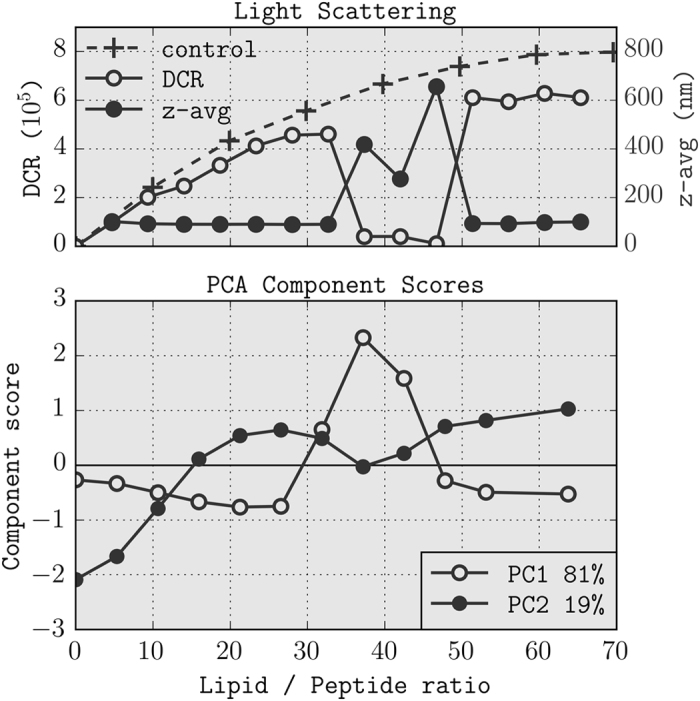
Comparison between light scattering and PCA decomposition of CD spectra. (**A**) Static and dynamic light scattering. The derived count rate (DCR) is a measure for the amount of backscattered light and is compared between a control curve of liposomes in buffer (black crosses) and a titration of vectofusin-1 with the same liposomes (open black circles). The estimated diameter of the particle calculated from dynamic light scattering is shown as the z-average in the same figure on the scondary axis (solid black circles), where the first datapoint is left out because the low scattering of peptide alone makes the computation of the z-average unreliable. (**B**) PCA scores as function of lipid/peptide ratio.

To follow structural changes and measure the binding of vectofusin-1 (sequence: KKALLHAALAHLLALAHHLLALLKKA-NH_2_) to POPC/POPS (3/1 mol/mol) liposomes, we titrated 30 *μ*M of peptide (0.1 mg/ml) by stepwise addition of liposomes from a 3.2 mM stock solution in 20 mM NaH_2_PO_4_ at pH 5, and recorded a circular dichroism spectrum for each step. The results of this titration are shown in Fig. 1A. Without liposomes, vectofusin-1 (VF1) is mostly unstructured (estimated 20% helix), as judged from the lineshape and the signal intensity at 222 nm. Upon addition of liposomes the peptide undergoes a structural change and becomes increasingly *α*-helical (lipid/peptide ratio 0–27). A further increase in the liposome concentration causes differential light scattering^21^, manifesting itself as a nonzero baseline best visible at 260 nm, and by the spectra no longer passing through the isodichroic point at 204 nm (L/P ratio 32–42). Interestingly, upon further addition of liposomes the light scattering disappears (L/P ratio 48–64). Similar behaviour has been observed before for the LAH4 peptide^11^ which exhibits potent antimicrobial and transfection activities, and is in agreement with the static light scattering experiments shown in Fig. 2A. The isodichroic point at 204 nm is consistent with a two-state transition from unstructured to *α*-helical^1^,^17^. Because the peptide structural changes have no effect on the signal intensity at this wavelength, it depends on the light scattering only. Unfortunately no second isodichroic point exists where the CD signal depends solely on the liposome-induced structural changes. We therefore set out to correct the spectra for the light scattering.

### Principal Component Analysis

The independence of scattering and structural changes should make it possible to decompose the spectra by principal component analysis^12^,^13^,^14^, a decomposition method based (usually) on singular value decomposition (SVD). PCA and SVD have been used extensively to decompose CD spectra into their structural constituents^6^ and are well established methods for the analysis of spectroscopic data^14^, but we found no reports where PCA or other decomposition methods are used to correct for light scattering.

PCA decomposition of the CD data in Fig. 1A, results in two components (Fig. 1B) that fully (99.88%) explain the variance in the data. The shape of the curves immediately suggests that the first component (81%) describes the light scattering and the second component (19%) the structural changes. The amount by which each of these components is present in the spectra (the PCA score) is shown in Fig. 2B as a function of lipid/peptide ratio. Samples with lipid/peptide ratios of 32–42 show changes in the first component, whereas samples with L/P ratios of 0–21 show an increase in the second component upon addition of liposomes (see also Fig. 1 in the Supplementary Information).

### Static and Dynamic Light Scattering

Comparison of Fig. 2A,B indicates good agreement between the first principal component and the amount of scattered light and particle size from static and dynamic light scattering measurements, confirming that it corresponds to the light scattering in the CD spectra. The second component resembles a sigmoidal association isotherm that reaches a plateau around a lipid–peptide ratio of 20 and describes the liposome-induced structural changes of vectofusin-1. The presence of a plateau indicates that further addition of liposomes does not cause increased peptide binding, therefore all peptides are bound to liposomes.

### Membrane Binding of Vectofusin-1

The sigmoidal shape of the association isotherm indicates that vectofusin-1 binding to POPC/POPS liposomes is most likely not a simple partition equilibrium as was observed for LAH4^11^ or melittin^9^. Based on these data alone however we do not wish to speculate on the exact nature of these differences, but it will be the subject of a further investigation.

## Discussion

The titration experiment started with peptide in solution to which the POPC/POPS liposomes were added in a stepwise manner. The dynamic light scattering data in Fig. 2A,B show that the amount of scattered light at the start of the titration is very close to the control experiment without peptide.

At lipid–peptide ratios around 35, a marked decrease in backscattering intensity is observed due to an increase in particle size. PC2 of the decomposed CD data indicates that under these conditions the amount of light scattering in the CD spectra increases. At a pH below the pKa of histidine, vectofusin-1 has a charge of +9. With 25% of negatively charged POPS lipids this leads to charge neutrality of the proteoliposomes at an expected lipid-peptide ratio of 9/0.25 = 36 if the negatively charged lipids on both leaflets are taken into account. Based on the good agreement between the theoretical and experimental value we hypothesise that negatively charged lipids from both leaflets are involved in the peptide binding.

When the lipid-peptide ratio is further increased, the backscatter decreases again and almost reaches the value of the control curve, indicating a decrease in particle size and thus reversibility of the proteoliposome self-association. This can only occur if vectofusin-1 binds reversibly and does not cause liposome disruption. Analysis of the light scattering during the CD titration indicates reversibility of the liposome self-association but not of the peptide structural changes. We interpret this as the peptides redistributing to the newly added liposomes, causing the lipid surface to regain part of its original negative charge, leading to dissociation (see also ref. 11). The coordinates of the PCA scores at low and high lipid/peptide ratio are close in space (Supplementary Figure 1), indicating a similar amount of light scattering before and after the proteoliposome self-association, and this is unambiguously confirmed by the autocorrelation curves from the dynamic light scattering experiments (Supplementary Figure 3). We also note the absence of liposome self-association at the beginning of the titration and interpret this as the proteoliposomes gaining a net positive charge, preventing aggregation.

**Figure 3:**
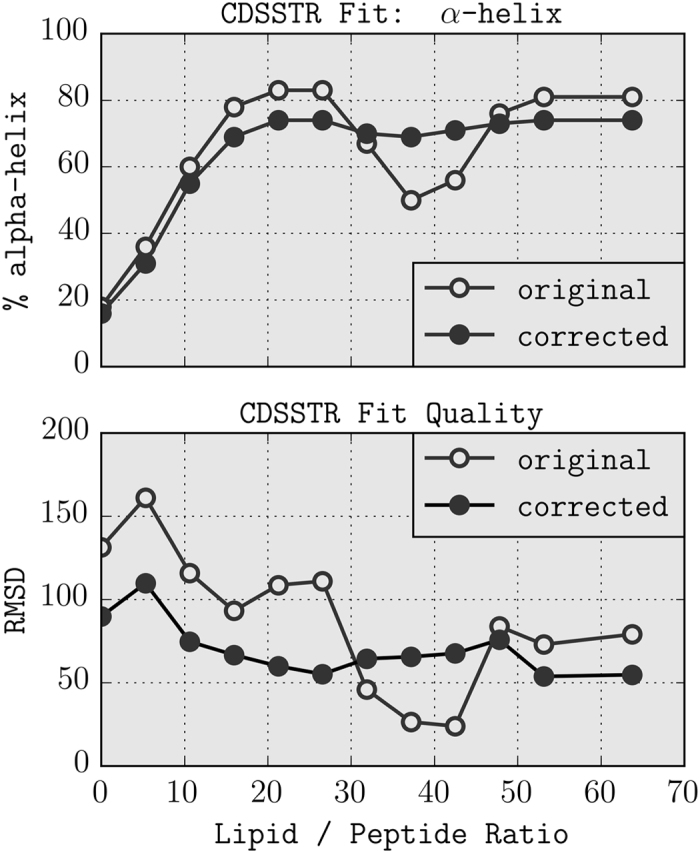
Secondary structure quantification by Dichroweb. (**A**) Percentage of *α*-helix as reported by the CDSSTR software. (**B**) Average RMSD of dichroweb fit, in units of 10^3^ deg · cm^2^ · dmol^−1^.

To quantify the secondary structure content, we compared the original CD spectra to those that were corrected for light scattering by removing the first principal component. After this correction the offset from the baseline at 260 nm nearly disappeared and all spectra pass through an isodichroic point at 204 nm (Supplementary Figure 2). For a fair comparison, the baseline offset of the original spectra was removed by subtracting the intensity at 260 nm from each spectrum (this appears to be common practice in the analysis of CD spectra, but it is not sufficient to remove the wavelength dependent differential light scattering). Spectra were then fitted using the CDSSTR method with reference set 4^23^, using the Dichroweb webserver^6^. Figure 3A,B summarise the results of this analysis, and further details are given in Supplementary Figures 4 and 5. It should be noted that the available reference sets are based on x-ray structures, and therefore not very suitable for peptides or proteins that contain significant amounts of disordered or P_II_ structure because of the absence of these structures in the reference sets. The estimation of the *α*-helical content however is generally considered to be reliable. Before the correction, a clear dip in the percentage of *α*-helix is observed, which reaches a minimum of 50% helix at L/P = 36. The DLS experiments indicate that this is a result of light scattering, which leads to underestimation of the helical content: after correction this dip nearly disappears and a continuous plateau at 75% *α*-helix is observed, corresponding to what might be expected for the binding of a cationic amphipathic peptide to a negatively charged liposome.

These results are in good agreement with biological studies indicating that vectofusin-1 strongly promotes virus-cell adhesion^15^,^24^. The lipid envelope of HIV-1 derived lentiviral vectors as well as eukaryotic cells (e.g. hematopoietic stem cells) have membranes containing negatively charged lipids^25^,^26^. We found that vectofusin-1 binds strongly but reversibly to mixed POPC/POPS liposomes and neutralises the membrane charges leading to liposome self-association at a very specific lipid/peptide ratio. In addition, the lack of vectofusin-1-induced membrane disruption may be an important factor that prevents this peptide from damaging viruses and cells, in contrast to other, very similar cationic amphipathic peptides that are known to cause membrane disruption (e.g. LAH4 and other antimicrobial peptides). Finally, the presence of an isodichroic point at 204 nm strongly suggests a structural change from unstructured to *α*-helical that can be described by a two-state model. To the best of our knowledge, this makes vectofusin-1 the first *α*-helical transduction enhancer.

## Conclusion

If light scattering in circular dichroism spectra is independent from structural changes associated with peptide-liposome binding, these spectra can be decomposed into principal components. In the experiment presented here, only two principal components were required to explain the variance in the data. One of these components is sensitive to light scattering only, and yields the same information as a separate static light scattering experiment. The other component depends only on peptide structural changes associated with liposome binding. In addition, removal of light scattering improves the quantification of the secondary structure content of vectofusin-1 and leads to more reliable results where the *α*-helical content is no longer underestimated, even in samples where light scattering causes a significant distortion of the CD spectra. The secondary structure of vectofusin-1 changes from mostly unstructured in solution to *α*-helical when bound to POPC/POPS liposomes. We conclude that PCA is a powerful method to remove and separately analyse the contribution of light scattering in circular dichroism data, increasing the amount of information that can be obtained from this experiment. We expect this method to be widely applicable and particularly useful in automated screening approaches; examples of expected applications are given in the last paragraph of the introduction.

Finally, we have shown that the transduction enhancing peptide vectofusin-1 causes reversible liposome self-association. This result is in excellent agreement with the increased virus-cell adhesion that is believed to be one of the major factors making vectofusin-1 such an efficient transduction enhancer. If the mechanisms described here and in the papers by D. Fenard^15^ and D. Ingrao^24^ are indeed the main factors underlying the vectofusin-1 efficacy as transduction enhancer, the method described here can be used in medium-throughput screening of peptide libraries for promising transduction enhancers by monitoring liposome self-association, liposome disruption and peptide structure and binding using a single CD titration.

## Methods

### Peptide Synthesis

The vectofusin-1 peptide was synthesised using standard FMOC solid-state chemistry on a Millipore 9050 synthesizer and purified by high-performance liquid chromatography on a Gilson reverse-phase HPLC with a Phenomenex perparative Luna C18-300 *Å*−5 *μ*m column with an acetonitrile/water gradient. After synthesis the peptide was washed three times with 4% (wt) acetic acid, freeze-dried, and stored at −18 °C. Its mass and purity were confirmed by MALDI-ToF mass spectromertry and HPLC. No impurities were detected.

### Circular Dichroism Spectroscopy

Lipids POPC/POPS (3/1 mol/mol) were mixed in chloroform, dried under a stream of nitrogen gas and kept under vacuum overnight, after which they were rehydrated with 20 mM NaH_2_PO_4_ at pH 5.0 and extruded 21 times through a 100 nm polycarbonate membrane. The liposome size distribution was a narrow Gaussian around 100 nm with a standard deviation of 9.6 nm, as measured by dynamic light scattering.

A solution of 200 *μ*l 20 mM NaH_2_PO_4_ containing 0.1 mg/ml of vectofusin-1 at pH 5.0 was titrated by adding lipid stock solution in steps of 10 *μ*l. The presence of 9 acetic acid counter ions was taken into account when calculating the peptide concentrations from the weight, and corrections for dilution during the titration were made. Circular dichroism spectra were recorded using a 1 mm quartz sample cell in a Jasco J-810 spectrometer at 25 °C. Datapoints were taken in steps of 1 nm with a scanning speed of 50 nm/min. The highest photomultiplier voltages observed during the titration ranged from 270 V at 360 nm to 520 V at 290 nm. A total of 10 spectra were acquired and averaged for each condition. A mock titration of lipids into 20 mM NaH_2_PO_4_ without peptide was used to generate control curves, which were subtracted from the CD-spectra of peptide-containing samples. The resulting intensities in millidegrees were converted to mean residue molar ellipticity (MRE = *θ*/10*nCl*, where *n* is the number of peptide bonds, *C* the concentration in M, and *l* the pathlength in cm).

### Static and Dynamic Light scattering

Aliquots of 1–12 *μ*l liposomes were added to 40 *μ*l, 320 *μ*M (1 mg/ml) peptide in a quartz cuvette that was kept at 25 °C during the experiment. Backscattered light was recorded at an angle of 173° on a Malvern Zetasizer Nano-S system with a 4 mW He-Ne laser (633 nm). Both static and dynamic light scattering were recorded. Static light scattering is represented in the paper by the derived count rate, a measure for the amount of scattered light corrected for the varying laser intensity which is automatically optimised for each sample. The particle sizes calculated from the dynamic light scattering autocorrelation curves are represented by the z-average, as reported by the Malvern software.

### Principal Component Analysis

The data analysis was carried out using the python programming language, version 3.4.2. PCA was implemented based on the SVD algorithm from numpy version 1.9.1, the source code and equations are given in the Supplementary Material. Before PCA, the spectra were mean-centered and auto-scaled. To correct the spectra for light scattering, the principal component that we assigned to light scattering was subtracted from the spectra, and although this led to a near-zero baseline at 260 nm we subtracted the small remaining baseline value from the spectra to make an equal comparison with the uncorrected spectra where the same baseline correction was applied. Although analysing the PC2 (structural) component alone would theoretically lead to a slight noise reduction by removal of higher order components, we opted not to do this to make a fair comparison with the uncorrected spectra. A detailed analysis of other multivariate methods is beyond the scope of this paper, but very similar results were obtained using PCA with a 5th degree polynomial kernel and PLS using the derived count rate from the static light scattering experiment as y-table, whereas independent component analysis and varimax rotation after PCA did not succeed in separating the light scattering from the structural changes.

### Quantification of Secondary Structure

The secondary structure of VF1 was quantified using the CDSSTR algorithm on the Dichroweb server, using reference set 4, with and without the correction for light scattering as described.

## Additional Information

**How to cite this article:** Vermeer, L. S. *et al*. Simultaneous Analysis of Secondary Structure and Light Scattering from Circular Dichroism Titrations: Application to Vectofusin-1. *Sci. Rep.* **6**, 39450; doi: 10.1038/srep39450 (2016).

**Publisher's note:** Springer Nature remains neutral with regard to jurisdictional claims in published maps and institutional affiliations.

## Supplementary Material

Supplementary Information

Supplementary Dataset 1

Supplementary Dataset 2
