# Supplementary material for: Simultaneous Analysis of Secondary Structure and Light Scattering from Circular Dichroism Titrations: Application to Vectofusin-1
Source: Sci Rep. 2016 Dec 22;6:39450. doi: 10.1038/srep39450 (PMC5177910; doi:10.1038/srep39450)
Supplement: Supplementary Information [file srep39450-s1.pdf]

SUPPLEMENTARY INFORMATION
Simultaneous Analysis of Secondary Structure and Light Scattering from Circular Dichroism Titrations: Application to Vectofusin-1.

Louic S. Vermeer, Arnaud Marquette, Michel Schoup, David Fenard, Anne Galy and Burkhard Bechinger

October 10, 2016

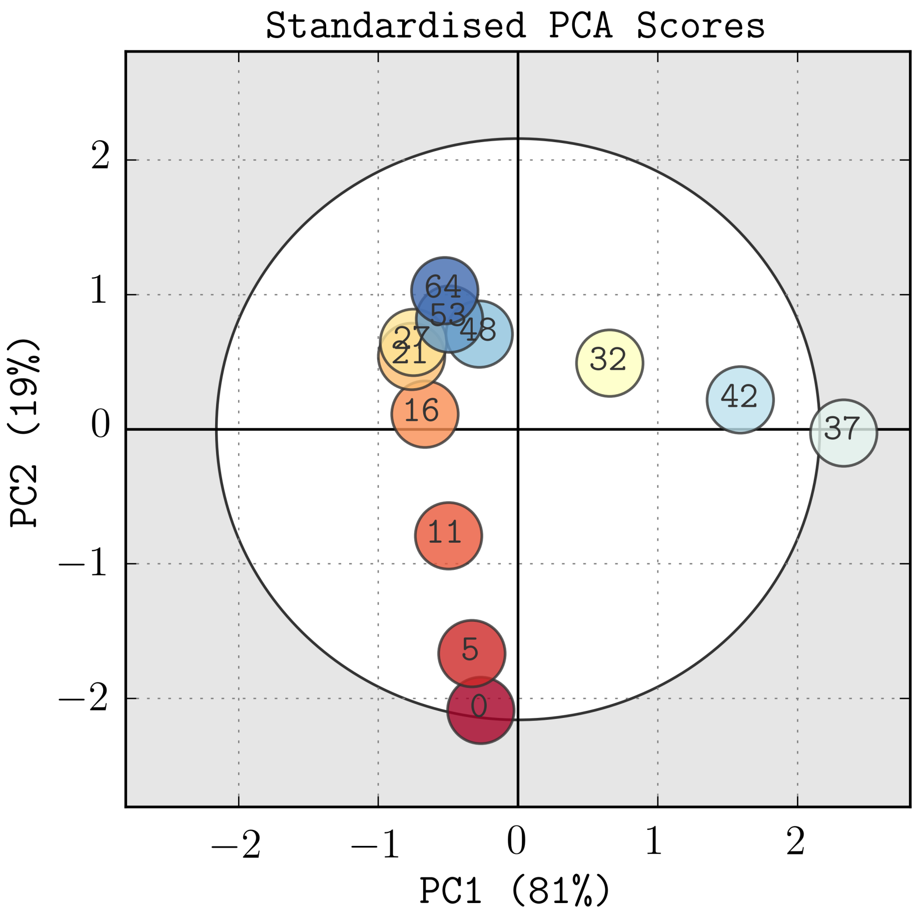

Figure 1: PCA scores of first and second component. Each lipid/peptide ratio is shown inside a circle with a colour corresponding to the spectra presented in the main paper. The values on the PC1 and PC2 axes represent the amount that each of these components is present in the sample. The independence of the two components is clearly visible by the nearly orthogonal scores of L/P ratio 0–16 on the one hand and 32–48 on the other hand. The reversibility of the proteoliposome aggregation is observed as clustering of the low and high L/P ratios. The confidence region drawn in white represents the 95% confidence region based on a beta distribution.

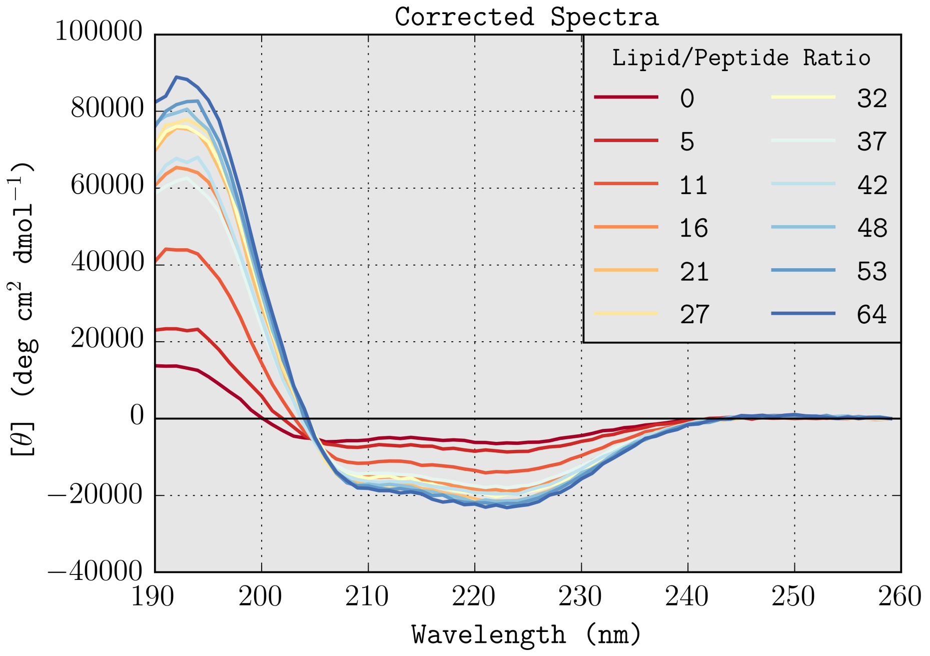

Figure 2: CD spectra after removal of scattering component.

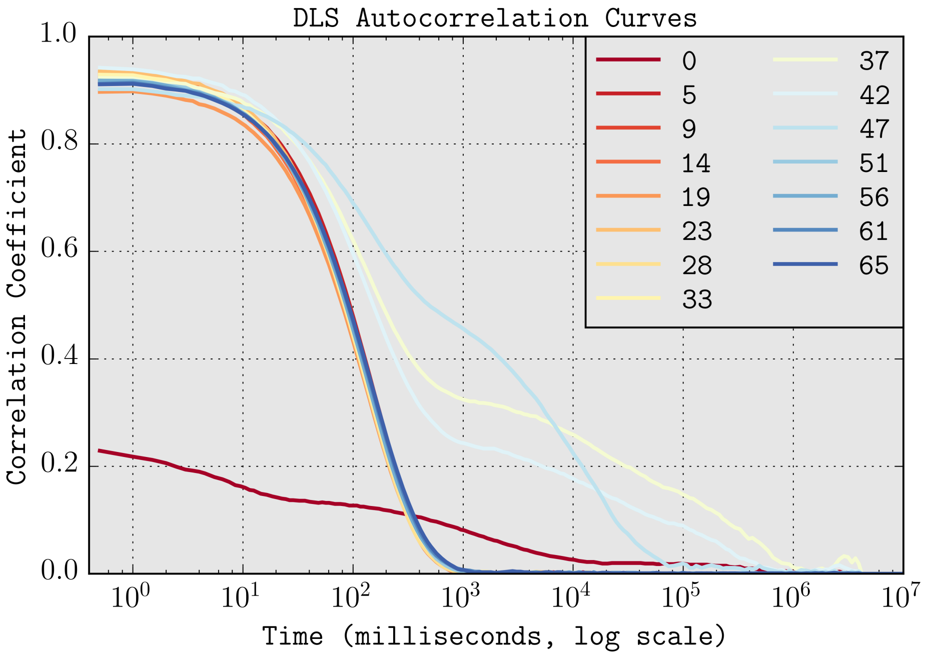

Figure 3: Autocorrelation curves of dynamic light scattering experiment. The first curve (labelled 0) is peptide alone and cannot be reliably analysed due to a low amount of scattered light. The presence of larger particles at L/P ratios of 32, 37 and 42 is clearly visible as the appearance of a "shoulder" with a larger correlation time. The return to smaller particle sizes is visible by the overlapping curves at all other L/P ratios tested.

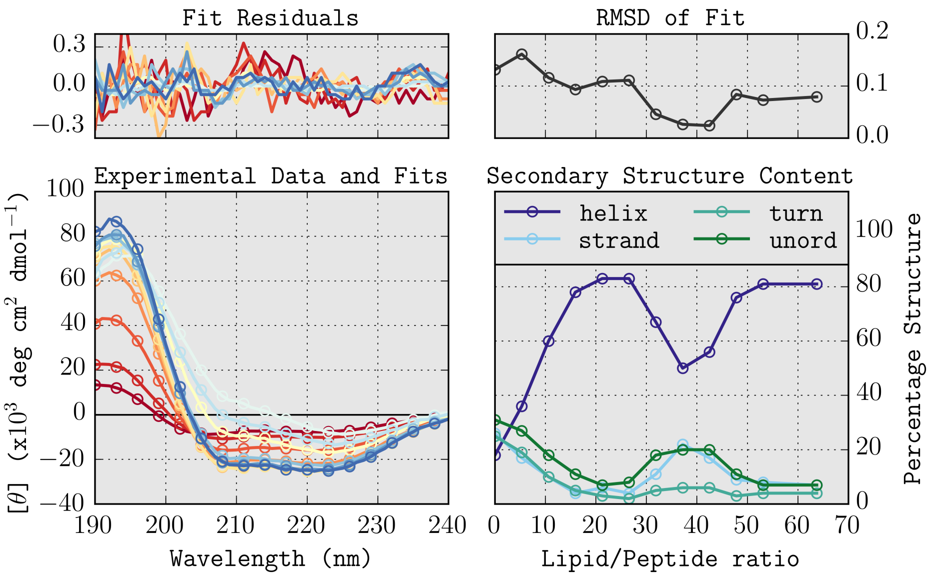

Figure 4: Dichroweb (CDSSTR) fit results for uncorrected spectra. The residuals and RMSD are given in the same units as the spectra.

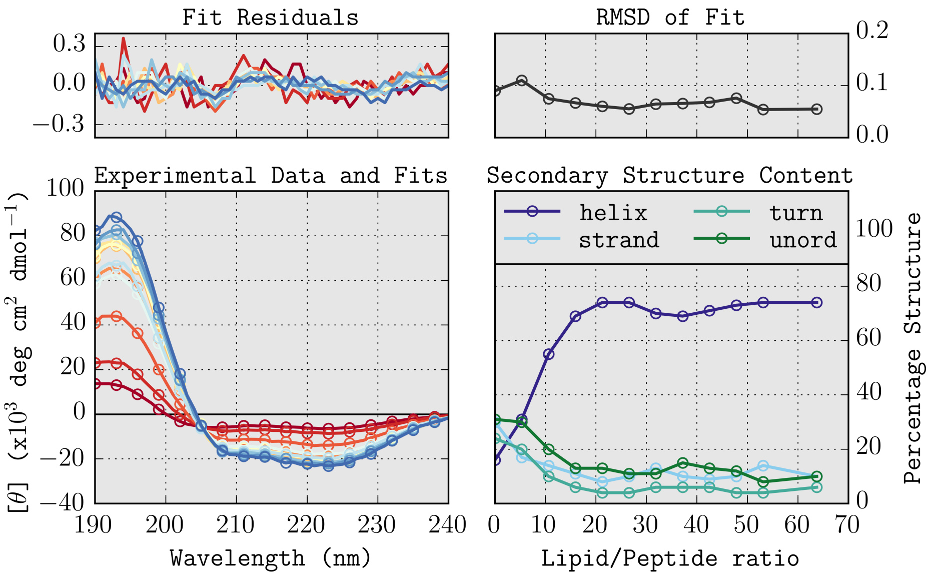

Figure 5: Dichroweb (CDSSTR) fit results for spectra after correction for light scattering. The residuals and RMSD are given in the same units as the spectra.

SVD-based PCA Decomposition of Titration Data.

The method and equations described below can be used to carry out principal component analysis [1, 2, 3] of any dataset. The specific application described in this paper is the separation of a light scattering from structural changes in CD spectroscopy, and is described in the final step, with additional details given in the rest of the paper.

- Record CD spectra [4, 5] and subtract baselines as usual. Do not apply any other data processing.
- Organise the spectra in an (n x m) data matrix X with elements x_{ij} where the n rows are the spectra (indexed by i) and the m columns are the wavelengths (indexed by j).
- Mean centre the data by subtracting the mean from each column:

$$\tilde{x}_{ij} = x_{ij} - \bar{x}_j \quad \text{with} \quad \bar{x}_j = \frac{1}{n}\sum_{i=0}^{n} x_{ij}$$

- Autoscaling: divide each column by its standard deviation [6].

$$\hat{x}_{ij} = \frac{\tilde{x}_{ij}}{s_j} \quad \text{with} \quad s_j = \sqrt{\frac{\sum_{i=0}^{n} (\tilde{x}_{ij} - \bar{x}_i)^2}{n-1}}$$

- Compute the singular value decomposition (SVD) of the centered and scaled data matrix using the built-in SVD function of scientific software. The SVD returns three matrces: U, S, and V^T

  Matlab, Octave, R, Python/Numpy, have built-in functions to compute the SVD. Example code is given in the supplementary information. Alternatively, you can use the built-in PCA function directly if there is one, taking care of the following: (1) some software uses different terminology (eigenvectors are sometimes called loadings) or scaling (scores and loadings may or may not be standardised), and (2) the PCA method should use a stable algorithm such as NIPALS [1] or SVD instead of eigenvalue decomposition.
- Compute the (standardised) scores T and loadings P.

$$T = U \cdot \sqrt{n-1} \quad \text{and} \quad P = (V \cdot S)/\sqrt{n-1}$$

- Compute the backscaled loadings [7]

$$\text{backscaled loadings} = P_{ij} \cdot s_j$$

- Analyse the scores (columns of T) and loadings (columns of P) as desired. See the main text of this paper for an example.
- To correct the data for light scattering, identify and remove the component k representing the light scattering.

$$\text{reconstructed data} = T_{k*} \cdot P_{k*}^T$$

  where the subscript $k*$ indicates that column k has been removed.

Source code for PCA

Note that all code for error handling and output was removed for clarity. Also note that we have chosen not to use built-in PCA methods to (1) illustrate the method and (2) be explicit about the data pre-processing and the definition of scores, loadings, and backscaled loadings. The python source code contains comments, but they are left out for the equivalent code provided for R and octave.

Python 3 (using Numpy)

```
import sys
import numpy as np

# get filename from command line and read data
# file format: samples in rows and variables in columns
fileName = sys.argv[1]
originalData = np.loadtxt(fileName)
n, m = originalData.shape

# mean centre and autoscale
X = originalData.copy()
Xmean = np.mean(X, axis=0)
X -= Xmean
Xstd = np.std(X, axis=0, ddof=1)
X /= Xstd

# compute singluar value decomposition
U, S, VT = np.linalg.svd(X, full_matrices=False)

# compute PCA matrices from SVD result
eigenValues = S**2 / (n - 1)
eigenVectors = VT.T
scores = U / np.sqrt(n - 1)   # equal to np.dot(X, eigenVectors)
loadings = eigenVectors * np.sqrt(eigenValues)
backscaledLoadings = loadings * Xstd
explainedVariance = eigenValues / np.sum(eigenValues)

# remove first component and reconstruct data without this component
removeComponent = 0
scoresNew = np.delete(scores, removeComponent, axis=1)
loadingsNew = np.delete(loadings, removeComponent, axis=0)
reconstructedData = np.dot(scoresNew, loadingsNew) * Xstd + Xmean
```

Octave / Matlab

```
fileName = argv(){1};
originalData = dlmread(fileName);
[n, m] = size(originalData);
```

```
X = originalData;
Xmean = mean(X, 1);
X .-= Xmean;
Xstd = std(X, 1);
X ./= Xstd;

[U, S, V] = svd(X, 'ECON');

eigenValues = diag(S.^2 ./ (n - 1))
eigenVectors = V.'
scores = U ./ sqrt(n - 1)
loadings = eigenVectors .* sqrt(eigenValues)
backscaledLoadings = loadings .* Xstd
explainedVariance = eigenValues ./ sum(eigenValues)

removeComponent = 1;
scoresNew = scores;
loadingsNew = loadings;
scoresNew(:, removeComponent) = [];
loadingsNew(:, removeComponent) = [];
reconstructedData = scoresNew * loadingsNew.'
reconstructedData = reconstructedData .* Xstd .+ Xmean
```

R

```
args = commandArgs(trailingOnly=TRUE)
fileName <- args[1]
originalData = read.table(fileName)
n <- dim(originalData)[1]
m <- dim(originalData)[2]

X <- originalData
X <- scale(X, center=TRUE, scale=TRUE)
Xmean <- attr(X, 'scaled:center')
Xstd <- attr(X, 'scaled:scale')

s <- svd(X)

eigenValues <- s$d^2 / (n - 1)
eigenVectors <- s$v
scores <- s$u / sqrt(n - 1)
loadings <- t(eigenVectors) * sqrt(eigenValues)
backscaledLoadings <- loadings * Xstd
explainedVariance <- eigenValues / sum(eigenValues)

removeComponent <- 0
scoresNew <- scores[,-1]
```

```
loadingsNew <- loadings[,-1]
reconstructedData <- scoresNew %*% t(loadingsNew)
reconstructedData <- t(reconstructedData) * Xstd
reconstructedData <- t(reconstructedData + Xmean)
```

References

[1] Svante Wold, Kim Esbensen, and Paul Geladi. Principal component analysis. *Chemometrics and Intelligent Laboratory Systems*, 2:37–52, 1987.

[2] S. W. Provencher and J. Glöckner. Analysis of the components present in kinetics (or titration) curves. *J Biochem Biophys Methods*, 7(4):331–334, Jul 1983.

[3] Victor A Shashilov and Igor K Lednev. Advanced statistical and numerical methods for spectroscopic characterization of protein structural evolution. *Chemical reviews*, 110(10):5692–5713, 2010.

[4] Norma J Greenfield. Methods to estimate the conformation of proteins and polypeptides from circular dichroism data. *Analytical biochemistry*, 235(1):1–10, 1996.

[5] Norma J Greenfield. Using circular dichroism spectra to estimate protein secondary structure. *Nature protocols*, 1(6):2876–2890, 2006.

[6] Robert A van den Berg, Huub CJ Hoefsloot, Johan A Westerhuis, Age K Smilde, and Mariët J van der Werf. Centering, scaling, and transformations: improving the biological information content of metabolomics data. *BMC genomics*, 7(1):1, 2006.

[7] Olivier Cloarec, Marc E Dumas, Johan Trygg, Andrew Craig, Richard H Barton, John C Lindon, Jeremy K Nicholson, and Elaine Holmes. Evaluation of the orthogonal projection on latent structure model limitations caused by chemical shift variability and improved visualization of biomarker changes in 1h nmr spectroscopic metabonomic studies. *Analytical Chemistry*, 77(2):517–526, 2005.
